# Calmodulin-Domain Protein Kinase PiCDPK1 Interacts with the 14-3-3-like Protein NtGF14 to Modulate Pollen Tube Growth

**DOI:** 10.3390/plants13030451

**Published:** 2024-02-03

**Authors:** Nolan Scheible, Paige M. Henning, Andrew G. McCubbin

**Affiliations:** School of Biological Sciences, Washington State University, Pullman, WA 99164, USA; nolan.scheible@wsu.edu (N.S.); phenning3@wisc.edu (P.M.H.)

**Keywords:** pollen tube growth, tip-growth, polarity, calcium-dependent protein kinase, 14-3-3 protein

## Abstract

Calcium-mediated signaling pathways are known to play important roles in the polar growth of pollen tubes. The calcium-dependent protein kinase, PiCDPK1, has been shown to be involved in regulating this process through interaction with a guanine dissociation inhibitor, PiRhoGDI1. To more fully understand the role of PiCDPK1 in pollen tube extension, we designed a pull-down study to identify additional substrates of this kinase. These experiments identified 123 putative interactors. Two of the identified proteins were predicted to directly interact with PiCDPK1, and this possibility was investigated in planta. The first, NtGF14, a 14-3-3-like protein, did not produce a noticeable phenotype when overexpressed in pollen alone but partially rescued the spherical tube phenotype caused by PiCDPK1 over-expression when co-over-expressed with the kinase. The second, NtREN1, a GTPase activating protein (GAP), severely inhibited pollen tube germination when over-expressed, and its co-over-expression with PiCDPK1 did not substantially affect this phenotype. These results suggest a novel in vivo interaction between NtGF14 and PiCDPK1 but do not support the direct interaction between PiCDPK1 and NtREN1. We demonstrate the utility of the methodology used to identify potential protein interactions while confirming the necessity of additional studies to confirm their validity. Finally, additional support was found for intersection between PiCDPK1 and RopGTPase pathways to control polar growth at the pollen tube tip.

## 1. Introduction

Pollen provides an excellent example of plant cells that use calcium as a second messenger to coordinate growth, and as they provide a relatively tractable model, they have been extensively used to study calcium signaling in plants (for review see [1]). Calcium concentrations in growing pollen tubes are dynamic, and pulses of increased free Ca^2+^ are followed by bursts of growth [2,3,4]. This effect is the result of the activation of a variety of calcium-sensing proteins that are regulated through binding of the cation. These proteins go on to influence downstream signaling events that coordinate growth, providing the cells with a finely tuned method for navigating the female floral tissues through which they must grow to deliver the sperm cells which they carry to egg cells [5].

An array of calcium-sensing proteins has been identified in pollen, including members of the calcineurin B-like interacting protein kinases (CIPKs), calmodulin-like (CML) proteins, calreticulin (CRT) and calcium-dependent protein kinases (CDPKs or CPKs) [6,7,8,9]. The latter group of enzymes has been the subject of a substantial amount of research in the last few decades. CDPKs have been implicated in pollen tube growth in diverse genera including Petunia, Zea and Arabidopsis. Two CDPKs were identified in Petunia pollen, and one was shown to modulate the polar extension of the tube because its over-expression resulted in swollen spherical tubes [10]. Evidence suggests that this kinase, PiCPDPK1, interacts with a rho guanine dissociation inhibitor (RhoGDI), a negative regulator of Rho GTPase activity, as their co-expression rescues the CDPK over-expression phenotype [11]. Rho GTPase activity is a well-characterized regulator of polar pollen tube growth in Arabidopsis [12]. In Zea, ZmCPK32 localizes to the plasma membrane, and its transient over-expression results in a decrease in both pollen germination and tube length [13]. The Arabidopsis genome contains 34 *CPK* genes, and 5 of these are preferentially expressed in pollen. Four isoforms, *AtCPKs 14*, *24*, *32* and *34*, when overexpressed result in significantly decreased tube length and increased tube width [14]. Two more, *AtCPK11* and *24*, have been reported to participate in the modulation of free K^+^ by decreasing the activity of an important membrane channel, SPIK [15]. Finally, *AtCPKs 2*, *6* and *20* have been shown to be crucial for maintaining the anion concentration at the pollen tube tip through activation of the anion channel SLAH3 [16,17]. It is clear that CDPKs/CPKs play an important role in pollen tube growth across diverse plant families, suggesting that this role is broadly phylogenetically conserved in the flowering plants.

We previously reported the identification of *PiCDPK1* and its interactor, *PiRhoGDI1*. However, kinases are known for their promiscuity of substrates [18,19], and to more fully understand the role of calcium and CDPKs in pollen tube growth, it is necessary to identify additional substrates of PiCDPK1. This “holistic” view is important for unravelling the role of calcium and its diverse intersection with other important signaling pathways.

Co-immunoprecipitation and subsequent mass spectrometry (Co-IP/MS) are powerful tools for identifying proteins of interest in interactor screens. Covalent bonding of an antibody to magnetic or agarose beads allows for the immobilization and thereby purification of an antigen-fusion protein of interest and complexes of proteins that interact with it [20]. Subsequent trypsin digestion of proteins eluted from the reaction provides short peptides that can be sequenced by mass spectrometry. The genes that encode them can then be identified, at least in species for which a proteome database is available. Co-IP/MS has advantages over other traditional protein interaction methods like Yeast 2-hybrid systems, because the interactions occur under natural cellular conditions and in the appropriate location in the tissue [21]. This technique has previously been used successfully to study protein interactions in pollen tubes. A study on the interactome of AtROP1 utilized GFP-tagged versions of the GTPase to pull-down the fusion protein and any interacting partners [22]. Using this approach, researchers identified 654 putative ROP1 interacting partners. Another study utilized a similar method to investigate the roles of nucleoside diphosphate kinases (NDPKs) beyond their function in nucleotide metabolism [23]. The findings supported roles for NDPKs in diverse biological processes that depend on nucleotide homeostasis. These studies demonstrated that Co-IP/MS studies can be effective and valuable tools for identifying interacting partners of proteins of interest, including kinases. Consequently, we sought to identify additional potential PiCDPK1 substrates using this approach.

Here, we report the identification of novel potential substrates of PiCDPK1 identified from a Co-IP/MS study using pollen lysates from stable transgenic plants over-expressing *PiCDPK1:GFP*. The genes encoding the pulled-down peptide sequences were identified in a tobacco proteome, and putative Arabidopsis homologs were filtered using criteria expected of proteins that interact with PiCDPK1. Further in silico analyses were used to predict direct interactors of PiCDPK1 from the list of filtered proteins, and two (*NtGF14* and *NtREN1*) were chosen for further study in vivo. Stable transgenic tobacco over-expressing GFP-tagged versions of NtGF14 and NtREN1 were generated in both wild-type and *PiCDPK1* over-expression genetic backgrounds. While *NtGF14* over-expression did not produce a phenotype in growing pollen tubes, it significantly rescued the *PiCDPK1* over-expression phenotype (spherical pollen tubes), providing additional support for in vivo interaction between these two proteins. *NtREN1* over-expression resulted in greatly diminished rates of tube germination; although, a small number of short, very-thin tubes managed to grow. This phenotype appears to be dominant over the *PiCDPK1* phenotype as pollen over-expressing both genes still largely failed to germinate. However, among the few tubes that managed to germinate and begin growing, those co-over-expressing both genes appeared to grow significantly longer than those over-expressing *NtREN1* alone.

## 2. Results

### 2.1. Affinity Purification and Mass Spectrometry Analysis Experimental Design

**Figure 1:**
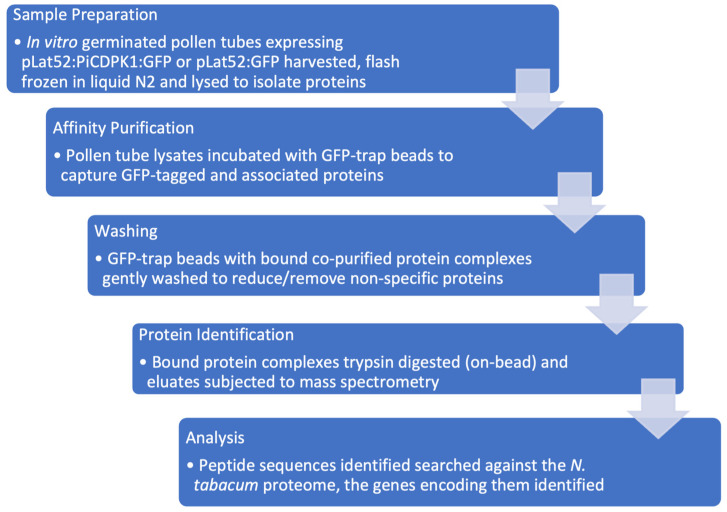
Proteomics workflow. Sample preparation: Pollen from transgenic *N. tabacum* over-expressing either *pLat52:PiCDPK1:GFP* or *pLat52:GFP* was collected and germinated in pollen germination media. Samples were flash frozen, pooled and total protein extracted. Affinity purification: pollen lysates were incubated with GFP-trap beads to bind to GFP-fusion and any interacting proteins. Washing: GFP-trap beads were washed to remove non-specific proteins. Protein identification: proteins captured on the GFP-trap beads were subjected to trypsin digestion and subsequent Mass Spectrometry analysis. Analysis: Peptide sequences were searched against the *N. tabacum* proteome to identify the genes that encode them. Contaminating peptides not derived from the tobacco genome were removed. Sequences were subjected to BLASTp to identify Arabidopsis homologs. Filtering: manual filtering to reduce/remove false positives was based on detection in at least two out of three biological replicates, enrichment > 1.75-fold in the treatment relative to the control sample, >1 unique peptide used for identification, and a putative sub-cellular localization consistent with a PiCDPK1 substrate (plasma membrane or cytosolic).

To further our understanding of the role of PiCDPK1 in the polar growth of pollen tubes by identifying additional substrates, we designed a pull-down experiment to enrich the proteins that interact with this protein kinase, which could then be identified by mass spectrometry analysis. Our goal was to identify native interactions in the endogenous, biologically relevant environment (Figure 1).

### 2.2. Identification of Putative PiCDPK1 Substrates

Following mass spectrometry analysis at the WSU Tissue Imaging, Metabolomics and Proteomics Laboratory (TIMPL), a total of 3012 proteins were identified in the treatment and control group samples. The sensitivity of this technology dictates that this list included false positives (including peptides present only in the control sample), and so, it was necessary to filter the data based on specified criteria. We first selected based on reproducibility, retaining only proteins that were present in at least two of the three biological replicates performed. Next, we chose to use the number of unique peptides recognized for each protein identification as a means of filtering to reduce the chance of miss-identification. Unique peptides are defined as peptide sequences that occur in only one specific protein sequence in a proteome and not others [24]. We chose a cut-off of at least two unique peptide groups for a protein candidate to be included in the final data set, in accordance with recent similar studies [22]. Lastly, we assigned a threshold level of enrichment for the proteins between the treatment and control samples. Common cut-offs for enrichment in previously published studies fall between 1.5× and 2× increases [25,26,27,28]. We chose 1.75-fold as the minimum increase in the PiCDPK1-GFP samples relative to GFP alone for inclusion on the list of potential candidates. This reduced the size of the dataset while including an isoform of the only previously identified substrate of PiCDPK1, a RhoGDI, *NtRhoGDI1* [11]. Applying the above criteria to the dataset filtered out 2817 proteins, leaving 195 candidate interactors.

The list of 195 proteins was then subjected to bioinformatic analysis. Arabidopsis has been more extensively researched than Nicotiana, and there are more bioinformatic tools available for this genus. To enable us to use these tools, we identified the closest Arabidopsis homologs of each candidate gene (using BLASTp) and used the information in their annotations as criteria to continue to refine our candidate list. PiCDPK1 localizes to the plasma membrane of pollen tubes and when over-expressed results in swollen spherical tubes, but removing the N-terminal acylation sites that target the kinase to the membrane abolishes both plasma membrane localization and the over-expression phenotype [10]. Hence, the function of PiCDPK1 is dependent on sub-cellular localization, and any bona fide substrates are expected to be localized either to the plasma membrane or the cytosol. Using the annotations of Arabidopsis homologs, we removed 72 additional proteins that were demonstrated or predicted to localize to cellular locations other than the plasma membrane or the cytosol. After applying the four different filters, our final set of putative PiCDPK1 substrates was reduced to 123. A subset of this annotated data set can be found in Table 1 (the full data set is available in Supplementary Table S1).

### 2.3. Bioinformatic Analysis of Filtered Putative PiCDPK1 Substrates

Proteins from the final data set were predicted to have diverse functions based on the annotations of their Arabidopsis homologs. Several have been reported to participate in activities important for pollen tube extension. For example, a homolog of Epsin 3, an accessory protein that facilitates vesicle biogenesis, identified in this study could potentially play a role in the deposition and recycling of wall materials at the tube tip, as Epsin 3 was shown to regulate vacuolar and secretory transport through the trans-golgi network in conjunction with another EPSIN protein [41]. Another protein, Pectate lyase 8 (PLL8), likely influences the pectin content as the up or down-regulation of a related family member alters the pectin content and vascular formation in Arabidopsis [37]. Pectin is a component of pollen tube walls, and at the pollen tube tip, it is the sole component [47], and hence, the pectin dynamics are important for growth responses in these cells. Perhaps a homolog of PLL8 affects the pectin content at the pollen tube tip to allow the tube to adjust its growth. Magnesium transporter 2 (MGT2) and MGT7 have been implicated in Mg^2+^ transport in leaf mesophyll cell vacuoles and general floral tissue, respectively [43,44]. As discussed above, ion transport and homeostasis are critical for germination and polarized tube extension, and these magnesium transporters could potentially be involved in maintaining the appropriate concentration of apical or sub-apical Mg^2+^ at different stages of pollen development. Further, a homolog of squalene epoxidase 3 (SQE3) was identified in this study, which in previous experiments has demonstrated a capacity to influence protein–lipid signaling by modulating the sterol composition, specifically affecting NADPH oxidases and reactive oxygen species (ROS) [46]. The homolog identified here could potentially affect sterol dynamics in the pollen tube wall, which in turn could impact factors like vesicle docking or the regulation of membrane-associated proteins.

Several protein candidates identified in this interaction study have been experimentally confirmed to participate in pollen tube growth. One, Vanguard 1 (VGD1), a pectin methylesterase, is hypothesized to modify the pollen wall and influence interactions between pollen with the female tissues [38]. Another, Sugar transport protein 10 (STP10), is expressed in pollen tubes and downregulated in the presence of glucose [42]. This suggests a role in sugar uptake, possibly in conjunction with other sugar signaling pathways. Of particular interest to this study, a homolog of Rho GTPase activating protein (RhoGAP), Rop enhancer 1 (REN1) was identified as being a candidate interactor of PiCDPK1. This RhoGAP participates in Rho GTPase signaling in pollen tubes by restricting active RopGTPase activity to the tube apex, and its localization is regulated by a phosphoglycerate kinase (PGK) [33,48]. It is possible that any or all of these proteins are involved in PiCDPK1 signaling in pollen tube growth. Phosphorylation by PiCDPK1 could potentially result in altering the activity or localization of these interactors or they could potentially be involved in the regulation of PiCDPK1. We next sought to gain additional evidence for select candidates to support their in vivo interaction with PiCDPK1.

**Figure 2:**
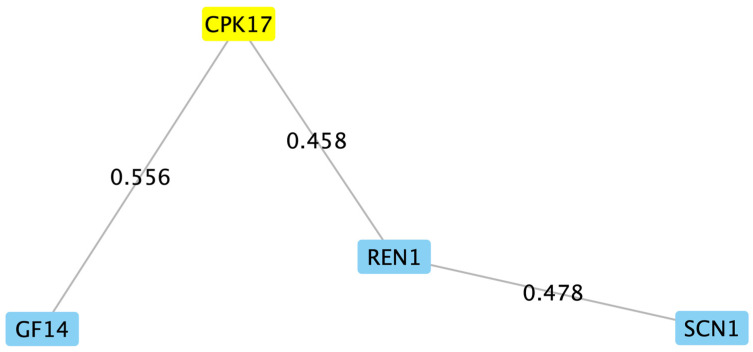
First neighbors of PiCDPK1. In our efforts to identify PiCDPK1 substrates, we performed a STRING analysis on the filtered list of candidates obtained from the Co-IP/MS experiment. The yellow highlighted box represents the Arabidopsis homolog (CPK17) of the gene of interest, PiCDPK1. The analysis predicted only two direct interactors of PiCDPK1, NtGF14 and NtREN1. A direct interaction was also predicted between NtREN1 and NtSCN1, RhoGDI. Node labels represent the proteins identified in the analysis, and edge labels represent the confidence score (scale 0–1) for the proposed interaction.

Unable (given the large number) to investigate all candidates, we performed a STRING analysis of the filtered list to identify candidates that might directly interact with PiCDPK1. STRING analysis utilizes data from known and predicted interactions to discern possible genetic networks [29], and we have previously found value in this method as a starting point from which to make testable hypotheses [49]. The analysis predicted only two direct interactors with PiCDPK1, NtGF14, a 14-3-3 protein, and NtREN1, a RhoGAP (Figure 2). We chose these two candidates to test hypotheses in vivo using stable transgenic tobacco plants.

### 2.4. Stable Over-Expression of NtGF14 in Tobacco Pollen Tubes

To further study the potential interaction between NtREN1 and NtGF14 and PiCDPK1, we transformed these genes into both a wild-type genetic background and a previously generated PiCDPK1 over-expressing line [11]. Both were placed under control of the pollen specific *Lat52* promoter [50] to avoid adverse effects on vegetative development. We further used GFP-tagged fusions of NtGF14 and NtREN1 to allow for the rapid identification of transgenics, as GFP fusions of these proteins have previously been reported to both fluoresce and retain biological function [48,51,52,53,54]. Use of GFP tags allowed for the easy identification of positive transgenic (T0) lines. In these plants, 50% of the pollen expressed GFP (reflecting a single active transgene insertion) and fluoresced when observed under confocal microscopy. These T0 plants were then self-fertilized, and the resulting seed was used to identify T1 progeny with 100% of pollen expressing GFP (lines homozygous for the transgene) to facilitate the quantitative assessment of transgene expression.

**Figure 3:**
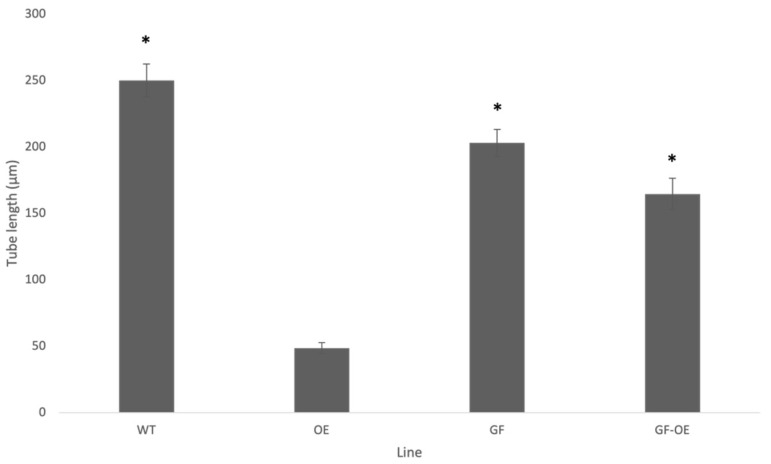
Average pollen tube length (μm) of wild-type or transgenic lines (as indicated) after 4 h incubation at 28–30 °C. Plant lines shown: WT (wild-type), OE (transgenic line expressing only PiCDPK1), GF (transgenic line over-expressing only NtGF14), GF-OE (transgenic line over-expressing both PiCDPK1 and NtGF14). Data are presented as means ± SE collected from >50 pollen tubes of each line. Asterisks indicate a significant difference between the samples labeled and the OE line (Student’s *t*-test, *p* < 0.001).

**Figure 4:**
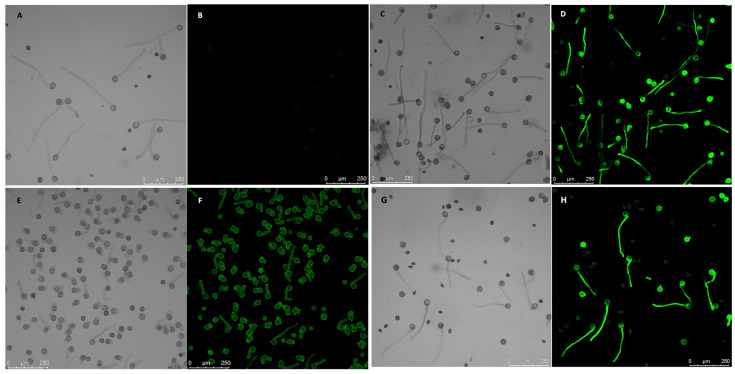
GF14 over-expression pollen phenotypes. Paired images show bright-field micrographs with equivalent fluorescence micrographs (black background). (**A**,**B**) Pollen tubes from wild-type tobacco; these extend in a polar fashion and do not fluoresce. (**C**,**D**) Pollen from transgenic tobacco homozygous for *pLat52:NtGF14:GFP*; there is no discernable morphological phenotype and tubes grow resembling the wild type (shown in (**G**)). (**E**,**F**) Pollen from transgenic tobacco homozygous for *pLat52:PiCDPK1:GFP* alone; ~100% of germinated pollen exhibit loss-of-polarity (visible as either spherical or unusually wide, short tubes). (**G**,**H**) Pollen from transgenic tobacco homozygous for both *pLat52:NtGF14:GFP* and *pLat52:PiCDPK1:GFP*; tubes are partially rescued and grow in a polar manner resembling wild-type tubes. Summary: NtGF14 does not produce a growth phenotype when overexpressed in pollen, but when co-expressed with PiCDPK1, it visibly rescues the ballooning, spherical tube phenotype associated with the over-expression of the kinase.

Pollen from T1 tobacco homozygous for the *pLat52:NtGF14:GFP* transgene cassette grew apparently normal tubes morphologically indistinguishable from those of the wild type (Figure 3 and Figure 4C,D). These tubes were, however, somewhat shorter than those of wild-type pollen grown for the same period (4 h). NtGF14 OE pollen tubes grew to an average length of 202.9 μm, 81% of the average tube length of 250 μm for wild-type pollen tubes (0.01 > *p* > 0.001) (Figure 3). This reduction in average tube length reflects a small difference in the rate of growth between the two genotypes. However, as we were able to readily identify lines that were homozygous for this transgene in T1 progeny, the difference in in vivo growth rate does not prevent pollen transmission of the transgene (3/10 homozygotes were identified for one independent line, 1/10 for another independent line).

We next investigated the effect of over-expressing *NtGF14* in the *PiCDPK1* over-expression background. It is hypothesized that the *PiCDPK1* over-expression phenotype, swollen or spherical tubes, results from the excess (calcium regulated) kinase activity of PiCDPK1 leading to an imbalance between phosphorylated and non-phosphorylated pools of endogenous substrates. We previously demonstrated that co-over-expression of one such in vivo substrate with *PiCDPK1* rescued the phenotype in tobacco pollen [11]. Here, we designed a similar experiment to determine whether co-over-expression of *NtGF14* with *PiCDPK1* affects the spherical tube phenotype. Transformants homozygous for stable over-expression of both *PiCDPK1* and *NtGF14* transgenes were generated as previously described [11]. By using plants homozygous for the *pLat52:PiCDPK1:GFP* as the tissue source for re-transformation with *pLat52:NtGF14:GFP*, we were able to identify T1 progeny lacking *pLat52:NtGF14:GFP* and homozygous for *pLat52:PiCDPK1:GFP* alone, as well as plants homozygous for both transgene cassettes. We used the former as control plants as they derived from tissues that experienced the same transformation and regeneration processes as the experimental lines. In the absence of the *pLat52:NtGF14:GFP*, pollen from plants continued to display the swollen and spherical tubes characteristic of the PiCDPK1 OE and grew to an average tube length of 48.6 μm. In contrast, however, the addition of *pLat52:NtGF14:GFP* caused pollen tubes to grow in a more polar manner to an average tube length of 164.6 μm, 339% of the PiCDPK1 OE pollen tube length (*p* < 0.001) (Figure 3). Compared to the average tube length of wild-type tubes (250 μm) and tubes over-expressing NtGF14:GFP alone (202.9 μm), the pollen co-over-expressing both PiCDPK1 and NtGF14 grew to average tube lengths of 66% (*p* < 0.001) and 81% (*p* > 0.01), respectively, of the aforementioned lines (Figure 3). This result suggested that the co-over-expression of *NtGF14* with *PiCDPK1* at least partially rescued the spherical tube phenotype observed in pollen over-expressing *PiCDPK1* alone (Figure 4G,H), supporting an in vivo interaction between these proteins.

**Figure 5:**
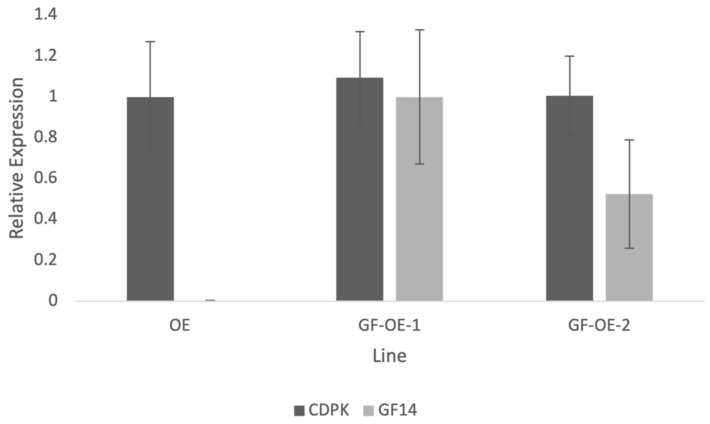
Quantitative PCR results showing expression of the PiCDPK1 transgene compared to the *NtGF14* transgene in pollen from stable transgenic tobacco lines. Plant lines shown: OE (transgenic line homozygous for *pLat52:PiCDPK1:GFP* alone) and GF-OE (two independent transgenic lines homozygous for both *pLat52:PiCDPK1:GFP* and *pLat52:NtGF14:GFP*). Results are normalized to the *Elongation factor 1-alpha* (*EF1*) internal tobacco reference gene, and the OE line is used as the control (expression level of 1.0). Data are presented as means ± SE from ≥3 biological replicates.

Using stable transgenic lines to investigate NtGF14 allowed us to measure transgene expression and validate this result by verifying that the partial rescue observed in the line co-over-expressing both *NtGF14* and *PiCDPK1* was not the result of silencing of the kinase transgene. We employed a similar method as previously described to confirm this result [11]. Designing primers that span the 5′ end of the *Lat52* promoter and the 3′ end of the gene of interest ensure that only the transgenes are being measured by negating any interference from endogenous tobacco homologs [11]. Expression of the *PiCDPK1* transgene was very similar between the OE (PiCDPK1 alone) and GF-OE (PiCDPK1 + NtGF14) lines, and the small difference measured was not significantly different (Figure 5). This result implies that the partial rescue of *PiCDPK1* over-expression by co-over-expression with *NtGF14* is the result of a bona fide interaction between the proteins in the pollen tubes and not a trivial result of decreased *PiCDPK1* expression.

### 2.5. Stable Over-Expression of NtREN1 in Tobacco Pollen Tubes

**Figure 6:**
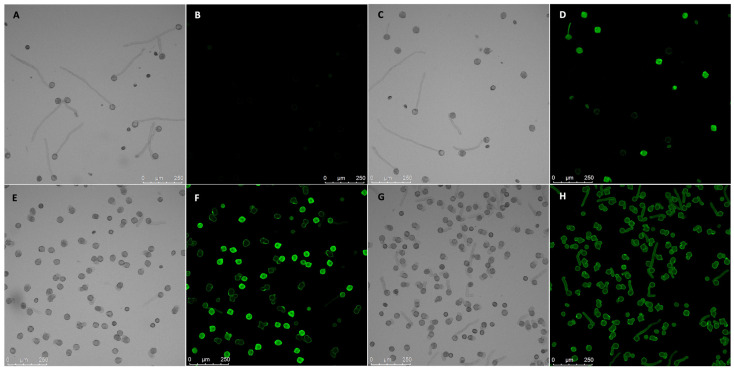
REN1 over-expression pollen phenotypes. Paired images show bright-field micrographs with equivalent fluorescence micrographs (black background). (**A**,**B**) Pollen tubes from wild-type tobacco; these extend in a polar fashion and do not fluoresce. (**C**,**D**) Pollen from transgenic tobacco heterozygous for *pLat52:NtREN1:GFP*; ~50% of pollen are fluorescing and do not germinate. (**E**,**F**) Pollen from transgenic tobacco heterozygous for *pLat52:NtREN1:GFP* and homozygous for *pLat52:PiCDPK1:GFP*; ~50% of pollen are fluorescing and do not germinate, while the other ~50% display the spherical PiCDPK1 OE pheno-type. (**G**,**H**) Pollen from transgenic tobacco homozygous for *pLat52:PiCDPK1:GFP* alone; ~100% of germinated pollen exhibit loss-of-polarity (visible as either spherical or unusually wide, short tubes). Summary: *NtREN1* over-expression results in greatly diminished germination of the pollen tube, and when co-expressed with *PiCDPK1*, the *NtREN1* phenotype is clearly dominant because pollen over-expressing both transgenes fail to germinate and produce a tube.

**Figure 7:**
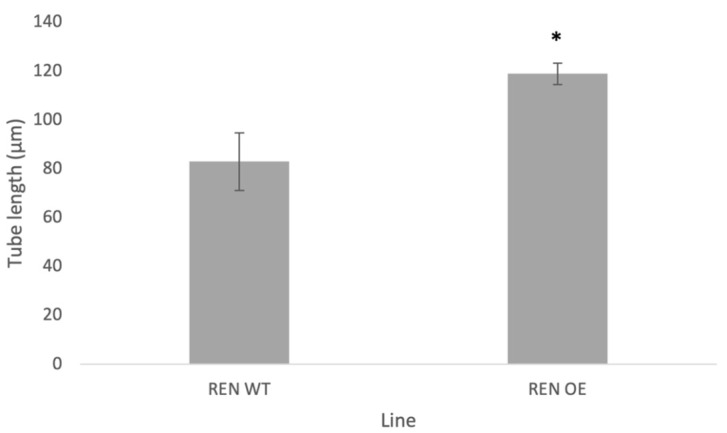
Average pollen tube length (μm) of transgenic lines (as indicated) after 4 h incubation at 28–30 °C. Plant lines shown: REN WT (transgenic line heterozygous for NtREN1 in the wild-type genetic background) and REN OE (transgenic line heterozygous for NtREN1 in the PiCDPK1 over-expression genetic background). Data are presented as means ± SE collected from >30 pollen tubes of each line. Asterisk indicates a significant difference between samples (Student’s *t*-test, *p* < 0.01).

We performed a similar set of experiments for *NtREN1*. We generated stable transgenic tobacco over-expressing *pLat52:NtREN1:GFP*. Pollen from T0 plants hemizygous for the *NtREN1* transgene were identified by the detection of GFP fluorescence. It was clear that ~50% of pollen was expressing GFP, but almost all failed to germinate. Dead pollen exhibits some autofluorescence, but the fluorescence observed was clearly not a result of death. The GFP fluorescing pollen grains had clearly hydrated (as evident by a swollen, round shape) and became metabolically active (GFP was being synthesized), but they failed to extend a tube. The small number that germinated (10–15%) produced very thin, short tubes (Figure 6D and Figure 7). Consistent with this observation, we were unable to identify a *NtREN1:GFP* homozygous line in T1 progeny generated from a heterozygous T0 self-fertilization. This result is consistent with a previous study with a different REN isoform, which reported that *REN4* over-expression in pollen tubes resulted in markedly decreased germination rates [53]. Further, the result is consistent with REN1 being a negative regulator of ROP, which is essential for pollen tube growth [33].

We next investigated the effect of co-over-expressing *NtREN1* with *PiCDPK1* as described above. Double transformants over-expressing both *pLat52:PiCDPK1:GFP* and *pLat52:NtREN1:GFP* were generated as previously described [11]. T0 transformants heterozygous for the *NtREN1* and *PiCDPK1* cassettes behaved in a similar manner as those in the wild-type background. Around 50% of pollen hydrated and displayed cytosolic GFP fluorescence (PiCDPK1 localizes to the plasma membrane) but lacked tube germination, while 50% displayed the typical PiCDPK1 OE spherical tube phenotype (Figure 6E,F) with fluorescence localized to the plasma membrane alone as expected for PiCDPK1-GFP. A small percentage of this pollen was able to germinate to produce short and skinny pollen tubes. Although little pollen germinated, those that did displayed a significant (*p* < 0.01) increase in tube length when compared to pollen over-expressing NtREN1 alone. Pollen co-over-expressing both *NtREN1* and *PiCDPK1* grew to an average tube length of 119 μm compared to the average tube length of 83 μm in pollen over-expressing *NtREN1* alone (Figure 7). Again, we were unable to identify a line homozygous for both *pLat52:PiCDPK1:GFP* and *pLat52:NtREN1:GFP* (0/20 plants screened). These results imply, unsurprisingly, that the PiCDPK1 OE phenotype is dependent on the presence of active ROP at the tube tip but do not provide support for PiCDPK1 being involved in the regulation of REN1 as no dramatic rescue or enhancement of the REN1-OE phenotype resulted from co-expression with PiCDPK1.

## 3. Discussion

In this study, we used a combination of co-immunoprecipitation and mass spectrometry (Co-IP/MS) to identify putative substrates of the pollen-specific kinase PiCDPK1. Using a GFP-fusion of the kinase, we were able to isolate interacting protein complexes from pollen lysate of stable transgenic tobacco over-expressing *PiCDPK1*. After filtering out false-positives, we compiled a list of 123 candidate interactors. Further bioinformatic analysis led us to proceed with additional studies on two of these candidate genes, *NtREN1* and *NtGF14*. First, we investigated these proteins in wild-type tobacco. Stable over-expression of *NtGF14:GFP* in tobacco pollen resulted in tubes that grow slightly more slowly than the wild type but display no visible morphological phenotype (Figure 3 and Figure 4C,D). Stable over-expression of *NtREN1:GFP* in tobacco pollen resulted in grains that hydrated and became active but did not germinate or extend a tube (Figure 6C,D). Co-over-expression of *NtGF14:GFP* with *PiCDPK1:GFP* partially rescued the spherical tube phenotype induced by expression of *PiCDPK1:GFP* alone, and tubes grow the resembling wild type (Figure 3 and Figure 4G,H). These results support NtGF14 being a novel bona fide interactor of PiCDPK1. Conversely, co-over-expression of *NtREN1:GFP* with *PiCDPK1:GFP* does not rescue the spherical tube phenotype because the tubes fail to germinate (Figure 6E,F). The REN1-OE phenotype is clearly dominant to that of PiCDPK1-OE. The percentage germination was low in both lines over-expressing REN1-OE and those co-over-expressing REN1 and PiCDPK1. If PiCDPK1 was involved in regulating REN1 directly, it seems likely that a pronounced phenotype would have been observed, particularly in regard to percentage germination, but none were observed. Hence, we conclude that this result does not support PiCDPK1 affecting the activity of REN1, either positively or negatively, or indeed for REN1 being an interactor of PiCDPK1. However, the small percentage of tubes co-over-expressing both NtREN1 and PiCDPK1 that germinated displayed a significantly increased tube length compared to those over-expressing NtREN1 alone (*p* < 0.01) (Figure 7). We do not believe this to be a result of direct interaction between the two proteins but rather an indirect result of PiCDPK1 activities. We previously reported that PiCDPK1 interacts and phosphorylates PiRhoGDI1, with the interaction leading to negative regulation of PiRhoGDI1 and recruitment of RopGTPase to the plasma membrane at the pollen tube tip [11]. It seems likely that the increased length observed in pollen tubes co-over-expressing NtREN1 and PiCDPK1 was caused by an increase in active ROP GTPase at the tube tip as a result of PiCDPK1-mediated release of cytosolic ROP GTPase from the RhoGDI protein. The effect of this would be to partially counteract the inhibition caused by NtREN1 by increasing the ROP GTPase pool at the tube tip.

Overall, these results confirm that the experimental design for the identification of protein interactors using co-IP/MS has the potential to reveal previously unknown interactions, but further investigations are critical to confirm that putative interactors in such data sets are “real”. The candidates identified as putative interactors of PiCDPK1 represent diverse protein families. Many come from families with proteins that have been experimentally implicated in pollen tube growth, including NtGF14 and NtREN1. NtGF14 is a 14-3-3-like protein, and members of this protein family are hypothesized to participate in diverse functions such as nuclear–cytoplasmic shuttling [55,56], regulation of membrane-bound ion channels at both the plasma membrane and tonoplast membrane [52,57,58], light perception [32,59], metabolism [60,61] and cell division or expansion [62,63]. The 14-3-3 proteins bind their client proteins and influence their activities by regulating translocation, structure, transcription and degradation [64]. The 14-3-3 proteins form homo- and heterodimers [65], and studies suggest they can interact with two distinct phosphorylated proteins at once [66,67]. The myriad of opportunities for interaction of 14-3-3 proteins with different binding partners point to an intricate and finely tuned network of cell regulation that is likely dependent on the specificity of expression in certain tissues at certain growth and developmental stages.

The regulation of 14-3-3 proteins themselves is also likely to affect their activities, and post-translational modifications, including phosphorylation, have previously been demonstrated [68,69]. In fact, phosphorylation of 14-3-3 proteins by CDPK/CPKs has been shown [70], supporting the proposed interaction presented here. A 2013 study identified an Arabidopsis 14-3-3 protein that interacts with a CDPK/CPK, AtCPK3, and phosphorylation of the 14-3-3 by CPK3 resulted in dissociation of the two proteins and subsequent degradation of the kinase [71]. In tobacco pollen, NtGF14 could be participating in any of the aforementioned processes and possibly even multiple processes. The regulation of membrane-bound ion channels at the plasma membrane is particularly attractive with regards to pollen tube growth [52,57,58], as several such channels have been reported to be key to this process [1].

Interestingly, a different 14-3-3 protein isoform identified was demonstrated to alter the localization of, and mediate the over-expression phenotype of, RhoGAP1 in tobacco pollen [72]. Pollen transiently expressing both the 14-3-3 and RhoGAP1 displayed a weakened sup-apical localization of the latter protein compared to pollen expressing RhoGAP1 alone. This co-expression partially rescued the RhoGAP1 over-expression phenotype of reduced germination and tube length [72]. Similar to the results presented here, over-expression of the 14-3-3 protein by itself did not produce a noticeable growth phenotype, but its co-expression with an interacting partner reduced the phenotype of the other protein [72] (Figure 4G,H). Intriguingly, a 14-3-3 protein and RhoGAP were also identified in this study, and it would be interesting in future studies to investigate the effect of co-over-expressing *NtGF14* and *NtREN1*. If a similar interaction is occurring, NtGF14 may also mitigate the *NtREN1* phenotype.

There are 15 different 14-3-3 protein isoforms in Arabidopsis and 17 in tobacco [73,74]. The many forms of this protein type suggest some functional redundancy among members, which makes knock-out or knock-down studies challenging [75]. We chose to utilize an over-expression strategy in the hopes of ascertaining the NtGF14 function based on the phenotype(s) observed. We were surprised not to see a substantial effect on tube growth morphology when NtGF14 was overexpressed, but this result may reflect the nature of the 14-3-3 protein function. The 14-3-3 proteins bind to phospho-serine or phospho-threonine peptide motifs in their targets [76,77], and it is possible that the over-expression of NtGF14 has no discernable effect on pollen tubes because its cellular targets are not phosphorylated (at least not at a high enough level), rendering the increased 14-3-3 protein inconsequential.

In our previous studies, we have demonstrated that co-expression of an in vivo substrate can rescue the phenotype induced by PiCPDK1 over-expression in pollen tubes, and we hypothesize this rescue is at least partially the result of titration of excess kinase activity keeping levels of phosphorylated to non-phosphorylated pools of substrates to more endogenous ratios [11]. The ability of NtGF14 to reduce the severity of the PiCDPK1 over-expression phenotype (Figure 3 and Figure 4G,H) suggests that a similar scenario may be happening here. Excessive amounts of the substrate provide an outlet for the overflow of kinase activity which reduces the over-phosphorylation of endogenous targets, thus alleviating the phenotype associated with the increase in phosphorylation. As previously mentioned, phosphorylation of a 14-3-3 protein by a CDPK/CPK resulted in the subsequent degradation of the kinase [71]. The partial rescue of the PiCDPK1 phenotype observed in this study could be the result of a similar process where co-over-expression with NtGF14 results in the degradation of PiCDPK1, but as we did not observe a discernable decrease in plasma membrane fluorescence associated with PiCDPK1-GFP, between tubes expressing PiCDPK1-GFP alone and those co-over-expressing PiCDPK1-GFP and NtGF14-GFP, this seems unlikely.

There are three REN isoforms in Arabidopsis [33], and all three have been implicated in polar expansion of cells like leaf epidermal cells and pollen tubes [33,48,78]. Here, we demonstrate that the over-expression of NtREN1 results in highly diminished rates of pollen germination, consistent with previous studies that made similar findings with RhoGAPs in Arabidopsis [53,79]. RhoGAP proteins function to stimulate the intrinsically slow hydrolysis of GTP by ROP GTPases, resulting in the biological inactivation of ROP activity [80]. It appears that increased RhoGAP activity prevents the pollen from starting tube growth (Figure 5), and this is most likely the result of a severe decrease in active ROP GTPase at any potential tube emergence site on the pollen grain. Without the downstream effects from active ROP GTPase signaling, the pollen is unable to coordinate important events that govern tube growth such as actin dynamics and vesicle trafficking, as well as maintenance of critical Ca^2+^ levels to activate Ca^2+^-sensitive signaling pathways at biologically appropriate times. RhoGAP over-expression inhibits ROP GTPase from becoming active, and as a result, pollen is unable to germinate. The same phenotype was observed in both the wild-type and PiCDPK1 over-expression genetic backgrounds, suggesting that the over-expression of NtREN1 nullifies the PiCDPK1 phenotype by preventing active ROP, the hypothesized culprit for the spherical PiCDPK1 phenotype [11], from ever becoming active, and hence, it is unable to accumulate at the tube apex and distort polar growth. Although these results do not support NtREN1 as being a substrate of PiCDPK1, the dominance of the NtREN1 over the PiCDPK1 phenotype provides further support that the latter phenotype is the result of increased ROP GTPase activity and supports a role of PiCDPK1 in the ROP GTPase regulatory pathway [11].

Calcium signaling is widespread in plant cells. Although a large amount of work has been carried out to identify the downstream signaling pathways stimulated by Ca^2+^, there is still much to learn about how plant cells interpret the Ca^2+^ code. The identification of Ca^2+^-activated enzymes and their downstream targets provide opportunities to attempt to unravel complex Ca^2+^-mediated signaling pathways. These different pathways are likely to overlap and display some functional redundancy which makes studying them challenging, but in order to obtain a more holistic view of pollen tube growth and Ca^2+^ signaling in general, it is crucial to understand the different cell processes governed and how they interact.

## 4. Materials and Methods

### 4.1. Plant Growth Conditions and Pollen Tube Germination

*Nicotiana tabacum* plants were grown in the Abelson Greenhouse at WSU at 25 °C with a 16/8 h photoperiod. Mature pollen was harvested from freshly dehisced anthers and cultured in pollen germination media (3 mM CaNO_3_·4H_2_O; 0.8 mM MgSO_4_•·7H_2_O; 1 mM KNO_3_; 1.6 mM H_3_BO_3_; 20 mM MES; 10% sucrose; pH 6.0) on a tilting platform for four hours at 28–30 °C to stimulate tube growth.

### 4.2. Protein Extraction and Affinity Purification

Stable transgenic tobacco over-expressing either PiCDPK1:GFP or free GFP were generated as previously described [11]. Pollen was collected from 50 flowers of tobacco expressing either *pPiCDPK1:GFP* or pGFP alone. Pollen was incubated in germination media for two hours at 28–30 °C to stimulate tube growth. After two hours of tube growth, samples were centrifuged at 2000 rpm for two minutes and the pellet was immediately frozen in liquid nitrogen. Pooled samples were ground in 1 mL of lysis buffer (25 mM HEPES pH 7.4, 10 mM MgCl_2_, 100 mM NaCl, 10% Glycerol, 1 mM DTT, 1 mM CaCl_2_ and 1% Triton-X 100 (Sigma Aldrich, St. Louis, MO, USA) and incubated for 30 min at 4 °C. Samples were centrifuged at 16,000× *g* for 10 min, and cleared lysate was added to GFP-trap magnetic beads (Chromotek, Planegg, Germany) and rotated end over end for two hours at 4 °C. Following magnetic decantation, beads were washed three times with wash buffer (lysis buffer supplemented with 0.5% Triton-X 100).

### 4.3. Sample Preparation for Proteomics Analysis by Liquid Chromatography-Mass Spectrometry

Proteins were digested “on-bead” using Pierce trypsin protease, MS grade (Thermo Scientific Cat. #90058 (Waltham, MA, USA)) as per the manufacturer’s instructions. Peptides were purified using G-Biosciences (St. Louis, MO, USA) C18 spin columns (Cat. #786-931) as per the manufacturer’s instructions. Dry residues were dissolved in a small volume of 3% acetonitrile in water with 0.1% formic acid. Peptide concentrations were determined using the Pierce Quantitative Colorimetric Peptide Assay (Thermo Scientific Cat. #23275). For each sample, 300 ng of peptides were used for proteomic analysis by liquid chromatography-mass spectrometry.

### 4.4. Proteomics Analysis by Liquid Chromatography-Mass Spectrometry

Analyses were performed using an Easy NanoLC 1000 UHPLC system coupled to a Fusion Orbitrap Tribrid mass spectrometer (ThermoFisher). The pre-column used was Acclaim PepMap 100 (75 μm × 20 mm, 3 μm particle size, Thermo Scientific Cat. #164535). The analytical column used was a Waters Peptide BEH C18 75 μm i.d. × 100 mm, 1.7 μm particle size (Waters Corp. (Milford, MA, USA) Cat. #. 186007481). Columns were equilibrated with 10 μL solvent A (0.1% formic acid in water) prior to sample loading. Samples were then injected onto the column in trapping mode in 10 μL volumes corresponding to 300 ng of total protein. The flow rate was kept at 350 nL/min (solvent A), and a gradient was applied with solvent B (0.1% formic acid in acetonitrile) over the following ranges, 2–35% B for 120 min, 35–90% in 6 min, and hold at 90% for 20 min.

A Fusion Orbitrap Tribrid mass spectrometer (ThermoFisher) was used for peptide MS/MS analysis. Samples were introduced using the Nanospray Flex (ThermoFisher) source. The positive electrospray mode voltage used was 2100 V, and the ion transfer tube was at 275 °C. Survey scans of peptide products were performed with a mass range of *m*/*z* 200–1800 at 120 K FWHM resolution. The AGC target was at 6^5^ and maximum injection time at 200 ms. The instrument was set to run at top speed mode with 3 s cycles for the survey and MS/MS scans. After the survey scan, tandem MS was performed on the most abundant peptides exhibiting a charge state from 2 to 6 of greater than 3000 intensity, by isolating them in the quadrupole at a window of 1.6 *m*/*z*. The AGC target was at 1^5^ and maximum injection time at 100 ms. The dynamic exclusion parameters employed were to be excluded after 1 time for 60 s with 10 ppm mass tolerance. HCD fragmentation was applied at 30% collision energy, and the resulting fragments were detected using the ion trap at a rapid rate. Raw data were processed using Proteome Discoverer v2.2 and searched against the *Nicotiana tabacum* proteome (downloaded from https://www.uniprot.org/ on 14 July 2021) using the SEQUEST HT engine.

### 4.5. Stable Over-Expression of NtGF14 and NtREN1 in Tobacco

Full-length coding sequences of *NtGF14* and *NtREN1* were amplified from a tobacco (*Nicotiana tabacum*) pollen cDNA library using the high-fidelity polymerase Accuzyme (Meridian Bioscience, Cincinatti, OH, USA) according to the manufacturers protocol with a slight modification. Primers were designed to amplify the full coding sequence of NtGF14, GF14-F (5′-atggcgtcgccacgcga-3′) and GF14-R (5′-ttactgctgctcattatctggttttgc-3′), and NtREN1, REN-F (5′-atgactaatcgcaatgctgatgct-3′) and REN-R (5′-tcatctcgagtttgtccttggag-3′). Purified PCR products were used as templates for Gibson reactions to generate the desired clones. To verify expression of transgenes and visualize transgenic pollen, fusion constructs were designed with each of the two transgenes being fused to GFP at the C-terminal. The Gibson reaction was used to generate *pGF14:GFP* and *pREN:GFP*, both driven by the pollen specific *Lat52* promoter. We used these constructs to generate stable transgenic tobacco (*Nicotiana tabacum*) using Agrobacterium-mediated plant transformation and tissue culture as previously described [11].

### 4.6. Analysis of Transformed Pollen Tubes

Confocal images were taken with a Leica TCS SP8 confocal laser-scanning microscope (Leica Microsystems, Wetzlar, Germany) at the 514 nm excitation wavelength to image GFP fluorescence. Light micrographs were also taken for each sample, and the pollen tubes were quantitatively analyzed by measuring tube lengths with the FIJI distribution of ImageJ v.214, as previously described [11].

### 4.7. Computational Analysis

BLASTp (version 2.12.0) [81] was used to identify Arabidopsis thaliana (annotation Araport11 8 March 2021) homologs of the *Nicotiana tabacum* proteins. Thresholds used to identify homologs were a percent identity of >30% and an e-value of <0.01. Protein–protein interaction networks were then predicted for the A. thaliana homologs using the Cytoscape (version 3.9.0) [82] STRING plugin (version 1.7.0) [83] with default settings. The networks were then clustered using the Cytoscape NCMine plugin (version 1.3.0) using a lenite clique threshold of 0.3. The resulting networks were uploaded from Cytoscape to the Network Data Exchange (NDEx) repository (version 2.5.2) [84] (UUIDs are listed in Supplementary Table S1). Identified genes were manually filtered based on the following criteria: >1.75-fold enrichment compared to the control sample (GFP alone), present in at least two out of three biological replicates, and >1 unique peptide being present for the gene. We also chose to use sub-cellular localization as a filter since the biological activity of PiCDPK1 is dependent on its plasma membrane localization [10]. Therefore, only candidate proteins predicted to localize to the plasma membrane or cytosol were included in the final data set.

### 4.8. Quantitative RT-PCR

RT-qPCR was performed at the WSU Genomics Core as previously described [21]. The forward primer used to amplify both the PiCDPK1 and NtGF14 transgenes was designed to span the 3′ end of the Lat52 promoter coding sequence, as described [21]. Reverse primers were designed to span the 5′ end of the PiCDPK1 coding sequence (5′-GCAGTGCTGATGGATTCTG-3′) and the 5′ end of the NtGF14 coding sequence (5′-CGTTCTTCGACGGTTAGTTC-3′). Control primers used for normalization were designed to amplify a small segment of the tobacco L25 ribosomal protein-coding sequence, as described [21], as well as an additional refence gene, Elongation factor -alpha (EF1): EF1-F (5′-TGGTGGTATTGACAACGT-3′) and EF1-R (5′-TGCAGTAGTACTTGGTGGTCTC-3′). Relative expression was determined using the 2^−ΔΔCT^ method [85].

## Figures and Tables

**Table 1:** The *N. tabacum* ID column displays the accession number identified from the *N. tabacum* proteome (UniProt). The Arabidopsis Homolog column shows the accession number and gene name of the Arabidopsis homolog obtained by BLASTp searches. Gene Family and Putative Function columns are based on information acquired from the Arabidopsis TAIR database. The first three proteins on the list represent those that are the focus of this study. The first (highlighted in orange) is PiCDPK1, which was the GFP-fusion protein used in the GFP-trap experiment. The second and third candidates in the table were obtained from a list of proteins that met the filtering criteria described above. These candidates were selected for further investigation based on the predicted interaction with PiCDPK1; the additional candidates represent members of the list of filtered proteins that were not specifically predicted to interact directly with PiCDPK1 by STRING analysis [29].

*N. tabacum* ID	Arabidopsis Homolog		Gene Family	Putative Function
	ID	Gene Name		
A0A1S4BR59	AT5G12180	*CPK 17*	Calcium Dependent Protein Kinase	Transduce Ca^2+^ signals to modulate pollen tube growth [30]
Q75ZD6	AT1G78300	*GF14 Omega*	14-3-3 protein	Facilitates interaction of actin-bundling plasma membrane proteins [31,32]
A0A1S3ZQG5	AT4G24580	*ROP1 enhancer 1*	Rho GTPase activation protein (RhoGAP) with PH domain	Inhibits activation of ROP1, regulates pollen tube growth [33]
A0A024AYA4	AT3G48700	*Carboxyesterase 13*	Carboxyesterase	Hydrolysis of esters [34]
A0A077DBK4	AT5G12020	*17.6 kDa class II heat shock protein*	Heat-shock protein	Interacts with and activates catalase in peroxisomes [35]
A0A0S0N4U0	AT5G19770	*Tubulin alpha-3*	Tubulin	Maintenance of growth against gravitational force [36]
A0A1S3X6C4	AT1G14420	*Pectate lyase-like 8*	Pectate lyase protein	Pectin synthesis/remodeling and secondary wall formation [37]
A0A1S3X7U8	AT2G47040	*Vanguard 1*	Plant invertase/pectin methylesterase inhibitor superfamily	Enhancer of growth of pollen tube in female floral tissues [38]
A0A1S3XSQ7	AT5G17820	*Peroxidase 57*	Peroxidase superfamily protein	Affects permeability of leaf cuticle [39]
A0A1S3XWB5	AT3G17390	*S-adenosylmethionine synthetase 3*	S-adenosylmethionine synthetase family protein	Involved in salt and H_2_O_2_ stress tolerance [40]
A0A1S3YE49	AT3G22530	Unknown	Hypothetical heat-shock protein	Unknown
A0A1S3Z0D6	AT1G34340	Unknown	Hypothetical alpha/beta-Hydrolase superfamily protein	Unknown
A0A1S3Z6E1	AT2G32730	Unknown	Hypothetical 26S proteasome regulatory complex	Unknown
A0A1S3Z926	AT3G59290	*Epsin 3*	Epsin family of endocytic proteins	Accessory proteins that facilitate vesicle biogenesis [41]
A0A1S4AFS9	AT3G19940	*Sugar Transport Protein 10*	Sugar Transport Protein	Glucose uptake in growing pollen tubes [42]
A0A1S4AKF1	AT1G16010	*Magnesium Transporter 2*	Transmembrane magnesium transporter	Tonoplast-targeted transporter for vacuolar accumulation of magnesium [43]
A0A1S4C178	AT5G09690	*Magnesium Transporter 7*	Transmembrane magnesium transporter	Low-affinity Mg^2+^ transporter [44]
A0A1S4CMX7	AT3G59990	*Methionine aminopeptidase 2b*	Methionine Amino-peptidase (MAP)	Required for development at various stages of Arabidopsis life cycle [45]
A0A1S4CPM1	AT4G37760	*Squalene epoxidase 3*	Squalene epoxidase enzyme	Plays a role in sterol biosynthesis in shoots [46]

## Data Availability

Data are contained within the article and Supplementary Materials listed above.

## Supplementary Materials

The following supporting information can be downloaded at: https://www.mdpi.com/article/10.3390/plants13030451/s1, Table S1: 1.75_networks_cluego_etc; Table S2: Proteomics data with filters.
